# Twelfth Biennial Meeting of the Italian Medical Association

**Published:** 1887-11

**Authors:** 


### EDITORIAL.

### TWELFTH BIENNIAL MEETING OF THE ITALIAN MEDICAL ASSOCIATION.

Not very many years ago the majority of the medical students from America who went abroad for advanced professional work or observation, were to be found in Paris. To-day, Vienna is considered by the same class of men as rather the most available place for general work; and it will generally be readily conceded that Germany and Austria together, present more and stronger attractions for American medical students in Europe than any other country, as they are of the foremost in the scientific medical investigation of the day. The preponderance of the work of this kind in these two nations has been so great, that some of their neighbors have not received due credit for what they have done and what they are doing. Among these is the kingdom of Italy. To those of our readers who are aware of the activity of this recently regenerated nation, only as it has shown itself in naval affairs and in Continental diplomacy, will find the account, given in another department of this number of the JOURNAL AND EXAMINER, of the twelfth biennial meeting of the Italian Medical society, both noteworthy and interesting. The letter published will show what good work and how much of it the Italian medical men are now doing. Careful reading of the letter shows, as our correspondent claims, that the Italians are active, energetic and intelligent workers in all branches of medical science; that in this as well as other departments of national life, awakening from their long period of inactivity, they are making rapid strides toward that proud position among the nations of the earth which their country once held.

